# PROTOCOL: Participation in organised sport to improve and prevent adverse developmental trajectories of at‐risk youth: A systematic review

**DOI:** 10.1002/cl2.1321

**Published:** 2023-04-03

**Authors:** Trine Filges, Mette Verner, Else Ladekjær, Elizabeth Bengtsen

**Affiliations:** ^1^ VIVE—the Danish Centre of Applied Social Science Copenhagen Denmark; ^2^ VIVE—the Danish National Centre of Applied Research Aarhus Denmark; ^3^ VIVE—the Danish Centre of Applied Social Science Aarhus Denmark; ^4^ Administration, The Danish National Centre for Social Research Copenhagen Denmark

## Abstract

This is the protocol for a Campbell systematic review. The main objective of this review is to answer the research question: What are the effects of organised sport on risk behaviour, personal, emotional and social skills of young people, who either have experienced or is at‐risk of experiencing an adverse outcome? Further, the review will attempt to answer if the effects differ between participants characteristics such as gender, age and risk indicator or between types of sport (e.g., team/individual, contact/non‐contact, intensity and duration).

## 1 BACKGROUND

### 1.1 The problem, condition or issue

Children and adolescents in the United States and in Europe spend more than half of their waking hours in leisure activities (Gracia et al., 2020; Larson & Verma, 1999; Wight et al., 2009). In the US, calculations from the 2003 to 2005 American Time Use Survey showed that youth aged 15–17 on average had 488 min (approximately 8 h) of leisure time per day, net of time spend in school, eating, grooming and doing household work (Wight et al., 2009). The corresponding numbers, using data from the time use surveys from Finland (2009–2010), Spain (2009–2010) and United Kingdom (2014–2015) were 571 min (approximately 9.5 h) for youth aged 10–17 (Gracia et al., 2020). For many, much of this time is spent either in unstructured peer focused activities or in front of the television, computer, and so forth. For youth aged 15–17 in the US, Wight et al., 2009 report that on average 57% of the 488 min of leisure time is spent this way. The corresponding number for European youth aged 10–17 is 50% of the time spend on computing programming, Internet use, computer games, watching TV, video watching and unstructured activities (Gracia et al., 2020). Some of this leisure time could probably be spent better; in ways that would both facilitate positive development and prevent the emergence of developmental problems (see Eccles & Gootman, 2002).

Leisure time activities such as organised sport is a good option as it provides young people with a valued place within a structured peer‐involved activity. In addition, sport is a voluntary activity that is both intrinsically and extrinsically motivating, and one that links young people to coaches who are positioned to assume the role of caring adult mentors. (Cronin & Allen, 2015; Petitpas et al., 2004) which in particular at‐risk youth may be in need of.

At‐risk youth may be defined as a diverse group of young, socially vulnerable people in unstable life circumstances, who are currently experiencing or are at risk of developing one or more serious problems such as school failure or drop‐out, mental health disorders, substance and/or alcohol abuse, unemployment, long‐term poverty, delinquency and more serious criminal behaviour (Arbreton et al., 2005; Quinn, 1999).

At‐risk youth often come from socio‐economically less advantaged and dysfunctional families (Treskon, 2016). At‐risk youth have often experienced at number of adverse events such as poverty, emotional or physical abuse and neglect, out‐of‐home placement, living with mentally ill or substance abusing parents and unstable housing situations. Thus, at‐risk youth often lack stable attachment figures and suitable adult role models.

Participation in organised sport have been shown to serve as important resources for reducing school failure and other problem/high‐risk behaviour (Parker, 2011). Larson (2000) argued that youth report more positive psychological states in voluntary structured activities, such as sports, because they experience challenge and perceive themselves to be active, in control, and competent in these settings. In addition, compared with other types of leisure activities, Larson et al. (2006) found that youth participating in sport reported significantly more experiences related to initiative, emotional regulation, and teamwork.

Also, the research by, for example, Camiré et al. (2009), Holt et al. (2009), and Holt and Neely (2011) has revealed relationships between involvement in sport and several positive outcomes for youths’ physical, cognitive, psychological, and social development. Additional examples include the ability to cope with pressure, communicate, receive feedback, set goals, solve problems, and deal with successes and failures (Papacharisis et al., 2005).

An example of an intervention based upon empowering is a Danish project engaging children aged 8–15 years old who have psychosocial challenges. The Danish NGO GAME initiated the project in 2018. The children were engaged in parkour activities in four cities in Denmark (Hansen et al., 2021). The intervention consist of 1 h of training for a period of 32 weeks. The training‐concept focus upon non‐competitiveness, social pedagogical principles, motivation, manageability and a recurring structure to insure successes for all participants. After this, a period of 8 weeks focusing at bridging barriers for the participants to participate in organised sports. The result shows that the participants gain motivation and act on this motivation for being physically active during their leisure time. One‐third of the participants became a lasting member of a sports organisation. Almost half of the participants also experienced gaining new friends (47%), and they strengthened their personal and social competences (Hansen et al., 2021).

Thus, while the benefits of youth sport participation have been of interest to sport researchers for some time and several systematic reviews are published, no research in the form of a systematic review with meta‐analysis to date has examined the benefits of sport for at‐risk youth in particular. It thus remains to be established to what extent sport participation have positive impacts on at‐risk youth.

A major difficulty in estimating causal effects of sport participation is the potential endogeneity of the young individual's life circumstance and developmental state that leads to the decision of participating in organised sport. It is thus very important to take self‐selection characteristics into account, or to quote Fullinwider (2006): ‘successfully control variables statistically to cut through the fog of correlation’ (p. 15).

Studies that simply assess the association between sport participation and developmental trajectories cannot be used to support conclusions about causation because these studies are not able to factor out selection effects and there is as little basis for denying the positive contribution of sports participation as there is for affirming it.

Hence, considering the fact that the population under investigation in this review by nature volunteer into the intervention, we believe it is vital that an appropriate comparison group and access to relevant pre‐tests is used to establish causality. Studies that control for important confounding factors provide some evidence for considering possible causal effects. While conclusions about causal effects must be very tentative, it is important to extract and summarise the best evidence available.

### 1.2 The intervention

The intervention of interest is organised sport. We will use the following definition of organised sport: a structured activity through an organisation, requiring physical exertion and/or physical skill and is generally accepted as being a sport (e.g., football, hockey, badminton, tennis, etc.). The common meaning of the term ‘sport’ is very wide and includes more disciplines than for example the Olympic definition. In Olympic terminology ‘sport’ refers to all events sanctioned by an international sport federation, and may comprise several disciplines of which not all are necessary Olympic disciplines (the list is constantly changing). Likewise, not all international sport federations are part of the Olympic programme but are members of the General Association of International Sports Federations (GAISF) and are contested at the World Games. Currently, there are 97 member‐Federations where several includes multiple disciplines (see https://gaisf.sport/members/).

By its nature organised sport is competitive, requiring the participants to develop personal discipline, setting goals, and striving to reach them and learn to sacrifice for delayed benefits. Generally there is a coach involved, from which participants follow directions and execute the skills taught. There are certain rules of engagement (regular participation, a certain number of days or hours of practice per week) which, of course, varies by sport, which can be individual or team oriented, contact sport, limited contact or no contact sport, and require different skills and competencies to perform effectively (strength, speed, dexterity, teamwork). The eligible setting is after‐school sport participation, that is, leisure activities, where social skills can be acquired through organised sport participation because of the unique demands of team sport such as the naturally afforded opportunities for youth to display skills such as co‐operation, compromise, teamwork, and leadership. But even participating in individual oriented sport, learning is an unavoidable part of the social life and participation in practice required when joining a sport club.

Sport clubs may be school or non‐school sport clubs as long as the activity takes place after school hours. In most European countries, the main means of promotion of sport are non‐school sports clubs, while in the USA (and to some extent Canada) school‐based sport clubs are the main providers of sport (Camiré, 2014; Laios, 1995). The popularity of non‐school sports clubs have however increased in the USA during the past couple of decades (Bennett et al., 2020).

### 1.3 How the intervention might work

Participation in organised sport have been shown to serve as important resources for reducing school failure and other problem/high‐risk behaviour (Parker, 2011). Youth report that they experience challenge and perceive themselves to be active, in control, and competent in these settings and compared with other types of leisure activities, report significantly more experiences related to initiative, emotional regulation, and teamwork (Larson et al., 2006; Larson, 2000).

There have been several strands of theories offered as a potential understanding of the theory of change behind sport participation (Holt et al., 2017; Jones et al., 2017).

For example, self‐determination theory (SDT) with its focus on the social‐contextual ingredients required for optimal growth and development (Ryan & Deci, 2000) is particularly useful when studying disadvantaged youth and have been extensively used as the theoretical framework guiding sport research (Jones et al., 2017).

SDT is a meta‐theory of human motivation and personality that addresses autonomous behaviours and conditions and processes that support voluntary engagement. Optimal functioning, development, and well‐being is achieved through the satisfaction of three innate psychological needs, namely autonomy, competence, and relatedness (Ryan & Deci, 2000). SDT is based on the assumption that a person's development, growth and wellbeing are supported to the extent these basic needs are accommodated by the social context. Autonomy is the desire to engage in activities of one's own choosing and the satisfaction of this need involves the experience of choice and the feeling that one is the initiator of one's own actions. Competence reflects the need to have an effect on the environment and to achieve desired outcomes and is fulfilled by the experience that one can effectively bring about desired effects and outcomes. Relatedness refers to the desire to feel securely connected to, understood and valued by others (Ryan & Deci, 2000).

Studies conducted in the sport setting have provided support for these basic tenets of SDT. With respect to the relationship of autonomy support (i.e., the coach is perceived as autonomy supportive by the athletes) to need satisfaction, research has shown that in the context of physical education, perceptions of an autonomy‐supportive climate were strong positive predictors of students’ perceptions of autonomy (Standage et al., 2003). In the same vein Ryan and Solky (1996) argue that social support may have positive psychological effects if the social support system satisfy one or more of the basic psychological needs SDT is built on, the need for relatedness in particular. The social support system in sports or the athletes’ perceptions of the social support on their team and by their coach may satisfy the need for relatedness. Research has shown that the team atmosphere created mainly by the coach has a strong influence on the social reality of athletes (Roberts & Treasure, 1992).

Regarding the need for competence, a dimension of the sport environment, which in particularly may satisfy this need is the coach's emphasis on athletes’ self‐referent improvement, mastery, and effort. A mastery environmental focus of the coach fosters perceptions of competence, because the self‐referenced criteria (e.g., effort) underlying competence judgements and ensuing feelings of success are more controllable and achievable compared to normative criteria (e.g., winning) according to Duda (2001).

Finally, the study by Reinboth et al. (2004); tested and found support for SDT's basic needs in the context of sport. The authors suggest that a social environment which is autonomy supportive, emphasises improvement and effort, and is socially supportive, may help maximise the satisfaction of sport participants’ basic needs which in turn may foster eudaimonic well‐being (well‐being achieved through experiences of meaning and purpose).

Another strand of theory which offers a potential understanding of the theory of change behind participation in organised sport is *Situated Learning* (Lave & Wenger, 1991). The term ‘situated learning’ refers to learning that occurs within a particular and authentic context through the individual's social participation. Rather than focusing on learning as a primarily cognitive process involving a number of tasks, situated learning theorists study the process in which individuals become new members of a learning community.

In their often cited work: ‘Situated Learning: Legitimate Peripheral Participation’, Lave and Wenger (1991); focus on acquisition of skills and knowledge that takes place outside traditional schooling within communities of practice. Lave and Wenger propose that learning should not be viewed as the mere transmission of knowledge but as a distinctly embedded and active process. Learning is perceived as a contextualised process in which content is learned through doing activities. Furthermore, Lave and Wenger suggest that motivation too is ‘situated’, as learners are naturally motivated by their growing value of participation (Lave & Wenger, 1991). Based on this approach children and youth participating in organised sport inherently become motivated to learn as this enables them to move from being novices to becoming full participants within the learning community. Reporting on a 3‐month ethnographic study conducted in a swimming club, Light (2010); explored the range of social, personal, and cultural development that occurs through children's participation in the practices of the club, drawing on Lave and Wenger's analytic concepts of situated learning and communities of practice. Light (2010); suggests that a range of important social learning, enculturation, and the development of identity arises from participation in the practices of the swimming club.

### 1.4 Why it is important to do this review

Although participation in organised youth leisure activities such as sport, have been shown to be associated with positive outcomes on general developmental indicators, such as school completion, employment and youth crime (Eccles et al., 2003; Parker, 2011), it is questionable whether the youth who would benefit most are those who chose to attend such programmes (Arbreton & McClanahan, 2002) or are given the opportunity to attend. It has been noted that the availability of such programmes is inequitably distributed across communities—with much lower availability in precisely those communities where the adolescents are at highest risk for poor developmental outcomes (Eime et al., 2015; Fullinwider, 2006; Owen et al., 2022). If there is limited or poor availability of quality facilities and activities in the local neighbourhood, transportation issues may be a barrier to attend organised sport.

And even if programmes are available they are typically not for free but comes with a participation fee and equipment costs out of reach for children living in poverty (Owen et al., 2022). According to Owen (2022) children and adolescents living in higher socioeconomic status households are 1.87 times more likely to participate in sport.

There is a need of strategies to increase the provision of sport opportunities, both facilities and affordability, in childhood and adolescence, to help develop and strengthen children and youths’ physical, cognitive, psychological, and social development through sport participation.

To the best of our knowledge there are currently no systematic reviews assessing what is known about the causal effects of sport participation on at‐risk youth.

We have located one systematic review on sport programmes for at‐risk youth, or as termed by the authors socially vulnerable youth (Hermens et al., 2017), however there were no restrictions on study design. The participant population was young people aged 10–23 who were socially vulnerable. Socially vulnerable is defined as: ‘Socially vulnerable youth represent a broad group, ranging from youth living in areas of low socioeconomic status (SES) to youth receiving residential care or non‐residential counselling. A common denominator is that they face stressors in their everyday life, such as income poverty, poor family management, low housing quality, and peers being involved in problem behaviour’ (p. 408). This definition of ‘socially vulnerable youth’ is in line with our definition of ‘at‐risk youth’. Studies published during 1990 to December 31, 2014 was included. Only studies that reported results on life skill development outcomes were included. As stated above, there were no restrictions on how the studies measured an impact, that is, qualitative studies as well as quantitative studies with or without comparison groups were included and some studies analysed one sport programme versus another sport programme. No meta‐analysis was performed, only a narrative analysis describing the studies and the results as stated in the studies.

We have located another systematic review including a broad range of physical activity programmes for participants aged 4–18 years considered to be at‐risk (Lubans et al., 2012). Participants with eating disorders and diagnosed psychiatric conditions were not eligible. The search was conducted on 21 December 2010 in six electronic databases (EMBASE, OVID MEDLINE, PsychINFO, PubMed, Scopus and SPORTDiscus) and conference abstracts, dissertations, theses and articles published in non‐peer‐reviewed journals were excluded. Quantitative studies with or without comparison groups reporting on social and emotional well‐being outcomes were included. No meta‐analysis was performed, only a narrative analysis describing the studies and the results as stated in the studies.

Further, we have located four systematic reviews on sport programmes (two of them including other physical activities as well) that did not restrict participants to be at‐risk youth.

The review by Eime et al. (2013); searched in June 2012 for studies reporting on the mental and/or social health benefits of sports programmes. They explicitly excluded studies or reports that addressed ‘exercise’, ‘physical activity’, ‘physical education’ or ‘recreation’, and not sport. Both quantitative and qualitative studies were included. After reviewing the included studies it was decided, that studies focusing on children and adolescents should be reviewed separately from studies focusing on adults and the review therefore focused on children and adolescents (18 or above). Only a narrative description of the studies were provided.

The review by Spruit, Assink, et al. (2016), included all studies examining the effect of physical activity interventions (including sports) on externalising and internalising problems, self‐concept, and academic achievement published before August 2015. Wilderness or adventure programmes, such as rock climbing, camping, backpacking, and hiking as a form of group therapy were excluded. The age range of the eligible samples had to be between 10 and 21 years old with a mean between 11 and 18. Only experimental studies (defined, as studies were a treatment group was compared to a comparison group of juveniles who did not participate in a physical activity intervention) were eligible. Finally, populations with physical health issues (except for obesity) were excluded. In total 57 studies were included of which 14 addressed sport interventions. A multilevel meta‐analysis for each of the four outcomes were performed showing overall small‐to‐moderate effects of physical activity interventions on all four outcomes. The moderating effect of whether the physical activity intervention consisted of sports or (aerobic) exercise activities only were analysed. Only the effect on one outcome differed between sport and (aerobic) exercise activities. Larger effects of physical activity interventions on self‐concept were found when the intervention consisted of (aerobic) exercise compared to sports intervention.

The (almost) same author team performed a systematic review with meta‐analysis on all studies addressing the relationship between sports participation and delinquency in juveniles which were published before October 2015 (Spruit, van Vugt, et al., 2016). Studies measuring sports participation combined with other types of activity participation and studies addressing sport interventions were excluded. The eligible age were reported as all studies with a mean between 12 and 18. Another eligibility criteria was that the study had to contain both athlete and non‐athlete samples, and both delinquent and non‐delinquent samples (or samples of the general population of adolescents), which seems a bit odd to base the study eligibility criteria's on the presence of the outcome in the samples. It was not required that the studies measured a causal relationship. In total 51 studies were included. A multi‐level analysis using correlation as the effect size was performed showing overall no correlation between sport participation and juvenile delinquency. The moderating effect of (amongst other things) the type of sport participation (team vs. individual, contact vs non‐contact and school setting vs. out‐of‐school setting) were analysed. One significant relationship was found, participants in individual sports were more delinquent than non‐participants whereas no relationship between participation in team sports and delinquency was found.

Finally, the review by Whitley and colleagues (Whitley et al., 2019), reviewed the research on sport‐based youth development interventions conducted within the US. The evidence of two types of interventions were searched for, a plus‐sport (i.e., sport adapted to maximise developmental objectives) intervention or a sport‐plus (i.e., sport used as a vehicle for development, with precedence on non‐sporting outcomes) intervention, published from 1995 through August 2017. Eligible programmes should be supplied to participants aged 10–24 years and data collected completely/partly in the US. Both quantitative and qualitative studies were included. In total 56 studies were included reporting on ten different interventions of which two were explicitly targeting youth from schools serving low‐income communities (Playworks) and at‐risk youth in various settings respectively (Doc Wayne). A narrative description of the results for each intervention were provided.

We specifically searched the Cochrane systematic reviews and located one marginally relevant for the current review (Ekeland et al., 2004). The review by Ekeland, 2004, searched in 2002 (month not reported) for studies reporting on exercise interventions for children and young people. The objective of the review was to determine if exercise interventions can improve self‐esteem amongst children and young people. Eligible activities included gross motor, energetic activity, for example, running, swimming, ball games and outdoor play of moderate to high intensity, or strength training. Only randomised controlled trials and quasi‐randomised trials, for example, those that use alternate allocation, date of birth, etc., were eligible study designs. Twenty‐three trials were included of which several included sport activities in the control condition. Three studies included at‐risk participants (children with learning disabilities (one study) and juvenile delinquents (two studies)), otherwise only healthy children and adolescents were included. A separate meta‐analysis was performed for these three studies, two of them included sport in the control condition.

Besides being up‐to‐date, a major difference between these five systematic reviews and the current proposal is that we will focus on sport programmes targeted at‐risk children/youth aged 6–18. We will only include studies with a control group. All relevant outcome areas will be analysed separately in a meta‐analysis taking into consideration the dependencies between effect sizes.

## 2 OBJECTIVES

The main objective of this review is to answer the research question: What are the effects of organised sport on risk behaviour, personal, emotional and social skills of young people, who either have experienced or is at‐risk of experiencing an adverse outcome? Further, the review will attempt to answer if the effects differ between participants characteristics such as gender, age and risk indicator or between types of sport (e.g., team/individual, contact/non‐contact, intensity and duration).

## 3 METHODS

### 3.1 Criteria for considering studies for this review

#### 3.1.1 Types of studies

The proposed project will follow standard procedures for conducting systematic reviews using meta‐analysis techniques.

Randomised and quasi‐randomised) controlled trials will be included. To summarise what is known about the possible causal effects of sport programmes, we will include all study designs that use a control group, that is, a group of children/youth not participating in organised sport. The control group may be offered no treatment or an alternative treatment.

The study designs we will include in the review are:

1. Randomised and quasi‐randomised controlled trials (allocated at either the individual level or cluster level, e.g., class/school/geographical area etc.).
2. Non‐randomised studies (participation has occurred in the course of usual decisions, the allocation to participation in organised sport and no participation is not controlled by the researcher, and there is a comparison of two or more groups of participants, that is, at least a treated group and a control group).

Studies using single group pre‐post comparisons will not be included. Non‐randomised studies using an instrumental variable approach will not be included—see Supporting Information: Appendix 1 (Justification of exclusion of studies using an instrumental variable (IV) approach) for our rationale for excluding studies of these designs. A further requirement to all types of studies (randomised as well as non‐randomised) is that they are able to identify an intervention effect. Studies where, for example, the treatment is offered to children in one school or community only and the comparison group is children at another school/community (or more schools/communities for that matter) cannot separate the treatment effect from the school/community effect.

#### 3.1.2 Types of participants

The review will include young people between 6 and 18 years of age, who either have experienced or is at‐risk of experiencing an adverse outcome such as school failure or drop‐out, substance and/or alcohol abuse, unemployment, long‐term poverty and delinquency/criminal behaviour.

At‐risk may be based on such indicators as the young person's level of association with negative peers (e.g., negative attitudes towards school and poor educational outlook, gang members, etc.), hanging out on the streets or in gang neighbourhoods, poor academic history, coming from a highly distressed or crisis ridden, low income family in a racially/ethnically segregated neighbourhood, and prior involvement in illegal and delinquent activities.

Studies where either the majority of participants are between 6 and 18 years of age or studies where a discrete age group within this range is provided will be included.

#### 3.1.3 Types of interventions

The intervention of interest is participation in leisure time organised sport. We will use the following definition of organised sport: a structured activity through an organisation, requiring physical exertion and/or physical skill and is generally accepted as being a sport. Generally there is a coach involved, from which participants follow directions and execute the skills taught. The organisation providing the activity may be school or non‐school sport clubs as long as the activity takes place after school hours. Leisure time physical activity defined as any unstructured physical activity outside of school hours is not eligible.

Traditional forms of sport provision are youth sport programmes designed to introduce participants to a specific sport that satisfies the desire for belonging, physical fitness, and fun. Quite different from traditional sport programmes are youth sport programmes that make an effort to teach sport skills and life skills concurrently containing clear expectations for achievement and learning. These programmes are also termed sport‐based youth development interventions (Petitpas et al., 2005). In these programmes sport is mostly considered a *necessary*, but not *sufficient* condition for the achievement of certain outcomes (Coalter, 2010).

Only the former type of programme is eligible, thus programmes in which sport is augmented with a parallel programme to maximise their potential to achieve certain developmental outcomes will be excluded. Also, multiple health behaviour intervention studies (e.g., co‐interventions such as a dietary programme combined with sport) will be excluded.

We will exclude studies that only address ‘exercise’, ‘physical activity’ or ‘physical education’, and not sport. In addition, we will exclude studies of yoga and studies of outdoor adventure programmes.

The comparison population are young people at‐risk who do not attend organised sport programmes.

#### 3.1.4 Types of outcome measures

##### Primary outcomes

The primary focus is on measures of problem/high‐risk behaviour, such as delinquency, drug and alcohol use, high levels of externalizing problems, school failure, and in the longer run employment, education, training (NEET status). These outcomes may be measured by self‐reports or reports by authorities, administrative files, registers.

##### Secondary outcomes

A secondary focus is on measures of personal, social and emotional outcomes.

Only valid and reliable outcomes that have been standardised on a different population (and is ‘objective’, i.e., not ‘experimenter‐designed’) will be included. Examples of valid outcomes are measures from the Social Skills Rating System (SSRS; Gresham & Elliott, 1990) or the revision of the SSRS, called the Social Skills Improvement System‐Rating Scales (SSIS‐RS; Gresham & Elliott, 2008), the Strength and Difficulties Questionnaire (SDQ; Goodman, 1997) and the Academic Competence Evaluation Scales (ACES) (DiPerna & Elliott, 1999).

Studies will only be included if they consider at least one of the primary or secondary outcomes. If it is not clear from the description of outcome measures in the studies whether they are standardised, we will use electronic sources to determine whether a measure is standardised or not. We will not consider measures where researchers have picked a subset of questions from a standardised measure.

It will be reported if any potential adverse effects have been evaluated in any included studies.

#### 3.1.5 Duration of follow‐up

Time points for measures considered will be:

- While actively engaged in organised sport
- At cessation of participation to 1 year after cessation
- More than 1 year after cessation

#### 3.1.6 Types of settings

The eligible setting is after‐school sport participation, that is, leisure activities. Public as well as private suppliers, including non‐profit organisations are eligible.

### 3.2 Search methods for identification of studies

Relevant studies will be identified through electronic searches in bibliographic databases, grey literature repositories and resources, hand search in specific targeted journals, citation tracking, contact to international experts and Internet search engines. A date restriction of 1970 and onwards will be applied.

#### 3.2.1 Electronic searches

The following electronic bibliographic databases will be searched:

- ERIC (EBSCO) 1970–2023
- Academic Search (EBSCO) 1970–2023
- EconLit (EBSCO) 1970–2023
- PsycINFO (EBSCO) 1970–2023
- SocINDEX (EBSCO) 1970–2023
- International Bibliography of the Social Sciences (ProQuest) 1970–2023
- Sociological Abstracts (ProQuest) 1970–2023
- Science Citation Index Expanded (Web Of Science) 1990–2023
- Social Sciences Citation Index (Web Of Science) 1990–2023
- EMBASE (OVID) 1974–2023

#### 3.2.2 Description of the search‐string

The search string is based on the PICO(s)‐model, and contains two concepts, of which we have developed two corresponding search facets: population characteristics and the intervention. The search string includes searches in title and abstract as well as subject terms and/or keywords for each facet. The subject terms in the facets will be selected according to the thesaurus or index of each database. Keywords will be supplied if the search technique provides additional results. Use of truncation and wildcards will be used to address English spelling variants.

#### 3.2.3 Example of a search‐string

Below is an exemplified search string utilised to search the database SocINDEX through the EBSCO‐platform. The search string is structured in the following order:

- Search 1–6 covers the intervention
- Search 7–18 covers the population characteristics
- Search 19 combines the two search facets

**EBSCO SocINDEX 1970**–**25 August 2022**. Search modes: Boolean/Phrase. Expanders: Apply equivalent subjects. Limiters: Date of Publication: 19700101–20221231

**Table:** 

#	Searches	Results
S1	TI sport* OR physical act* OR exercise*	13,136
S2	AB sport* OR physical act* OR exercise*	45,380
S3	SU sport* OR physical act* OR exercise*	18,703
S4	S1 OR S2 OR S3	51,526
S5	(DE ‘SPORTS ‐‐ Social aspects’) OR (DE ‘EXERCISE ‐‐ Social aspects’)	429
S6	S4 OR S5	51,526
S7	TI youth OR adolescen* OR young people OR young person* OR young adult* OR teens OR teenager* OR boy* OR girl* OR student*	130,732
S8	AB youth OR adolescen* OR young people OR young person* OR young adult* OR teens OR teenager* OR boy* OR girl* OR student*	312,730
S9	SU youth OR adolescen* OR young people OR young person* OR young adult* OR teens OR teenager* OR boy* OR girl* OR student*	177,403
S10	S7 OR S8 OR S9	354,251
S11	DE ‘YOUTH’ OR DE ‘YOUNG adults’ OR DE ‘YOUNG men’ OR DE ‘YOUNG women’ OR DE ‘ADOLESCENCE’ OR DE ‘TEENAGERS’	43,507
S12	S10 OR S11	355,582
S13	TI vulnerab* OR ‘at risk*’ OR ‘at‐risk*’ OR disaffect* OR ‘youth work*’ OR ‘youth care*’ OR ‘social work*’ OR ‘social care*’ OR underserv* OR depriv* OR minorit* OR ‘low SES’ OR ‘school dropout*’ OR ‘school failure*’ OR disadvantage* OR traumati#* OR maginali#ed OR ‘disruptive behavio#r’ OR abus* OR poverty OR crim* OR homeless* OR street* OR detach* OR delinquen*	204,265
S14	AB vulnerab* OR ‘at risk*’ OR ‘at‐risk*’ OR disaffect* OR ‘youth work*’ OR ‘youth care*’ OR ‘social work*’ OR ‘social care*’ OR underserv* OR depriv* OR minorit* OR ‘low SES’ OR ‘school dropout*’ OR ‘school failure*’ OR disadvantage* OR traumati#* OR maginali#ed OR ‘disruptive behavio#r’ OR abus* OR poverty OR crim* OR homeless* OR street* OR detach* OR delinquen*	480,590
S15	SU vulnerab* OR ‘at risk*’ OR ‘at‐risk*’ OR disaffect* OR ‘youth work*’ OR ‘youth care*’ OR ‘social work*’ OR ‘social care*’ OR underserv* OR depriv* OR minorit* OR ‘low SES’ OR ‘school dropout*’ OR ‘school failure*’ OR disadvantage* OR traumati#* OR maginali#ed OR ‘disruptive behavio#r’ OR abus* OR poverty OR crim* OR homeless* OR street* OR detach* OR delinquen*	320,643
S16	S13 OR S14 OR S15	595,767
S17	DE ‘AT‐risk youth’ OR DE ‘SCHOOL dropouts’ OR DE ‘SCHOOL dropout prevention’ OR DE ‘YOUNG people not in education, employment, or training’ OR DE ‘SOCIOECONOMICALLY disadvantaged students’ OR DE ‘POOR youth’ OR DE ‘STREET youth’ OR DE ‘HOMELESS youth’ OR DE ‘DELINQUENT youths’	2237
S18	S16 OR S17	595,825
S19	S6 AND S12 AND S18	2968
S20	S6 AND S12 AND S18 ‐ *Limiters ‐ Date of Publication: 19700101‐20221231*	2864

#### 3.2.4 Searching other resources

##### Hand‐Search

We will conduct a hand search of specific journals, to make sure that all relevant articles are found. The hand search will focus on editions published between 2020 and 2022 to secure recently unpublished articles which have not yet been indexed in the bibliographic databases. We will decide upon which journals to hand search based on the identified records from the electronic searches. The following are examples of specific journals which we may decide to hand search:

- Children and Youth Services
- The Future of Children
- Research on Social Work Practice
- Journal of Prevention and Intervention in the Community

##### Searches for unpublished literature in general

Most of the resources searched for unpublished literature include multiple types of references. As an example, the resources listed to identify reports from national bibliographical resources also include working papers and dissertations, as well as peer‐reviewed references and documents from preprint databases. In general, there is a great amount of overlap between the types of references in the chosen resources. The resources are listed once under the category of literature we expect to be most prevalent in the resource, even though multiple types of unpublished/published literature might be identified in the resource.

Further resources for identifying dissertations might be added during the search process. A final list of resources will be included in the appendix of the review.

##### Search for dissertations

We will search the following resources for dissertations:

- EBSCO Open Dissertations (EBSCO‐host)
- OATD—Open Access Theses and Dissertations (oatd. org)

##### Search for working papers/conference proceedings

We will search the following resources for working papers/conference proceedings:

- American Institutes for Research (AIR): https://www.air.org/
- *Manpower Demonstration Research Corporation (MDRC)*: https://www.mdrc.org/
- Urban Institute: https://www.urban.org/
- Google Scholar—https://scholar.google.com
- Google—https://google.com

##### Search for preprints/post prints/working papers

We will search the following resources for preprints, post prints, working papers and published papers:

- SportRxiv—Open access subject repository of preprints, post prints within sport, exercise, performance, and health research—https://sportrxiv.org/
- SocArxiv—Open archive of the social sciences of working papers, preprints, and published papers—https://osf.io/preprints/socarxiv
- PsyArXiv—Open archive of preprints, working manuscripts, and post prints in the psychological sciences—https://psyarxiv.com
- OSF Preprints—Open source multidisciplinary preprints as well as post prints and working papers—https://osf.io/preprints/

##### Search for reports

Public/Private Ventures (P/PV) publications (although Public/Private Ventures (P/PV) has ceased operations, its publications are archived with the Foundation Center's IssueLab: https://ppv.issuelab.org

##### Search for non‐US literature

We will search the following resources for non‐US literature:

- Danish National Research Database—http://www.forskningsdatabasen.dk/en
- SwePub—Academic publications at Swedish universities—http://swepub.kb.se/
- NORA—Norwegian Open Research Archives—http://nora.openaccess.no/
- CORE—research outputs from international repositories—https://core.ac.uk/
- Skolporten—Swedish Dissertations—https://www.skolporten.se/forskning/
- DIVA—Digital Scientific Archives—http://www.diva-portal.org/smash/

##### Search for systematic reviews

If we identify relevant systematic reviews during the search process, they will be used for citation‐tracking, to extract relevant references from the review.

##### Citation‐tracking

We will use citation‐tracking methods to identify more relevant literature. We will citation‐track forwards (by using Google Scholar and Web of Science) and backwards (by screening citations in the most relevant literature).

##### Contact to experts

We will contact international experts to identify unpublished and ongoing studies

##### Other criteria

Studies will not be excluded based on publication status or language (although the ability to assess the relevance of studies is limited by the language skills in the review team). Studies authored before 1970 will not be included.

### 3.3 Data collection and analysis

#### 3.3.1 Description of methods used in primary research

Randomised controlled trials are eligible, but we expect that a certain amount of studies will be conducted without randomisation of participants. Studies of the effect of participation in organised sport are required to have a control group for inclusion in the review. Participants may be allocated by, for example, time differences, location differences, decision makers, policy rules or participant preferences. We expect that the primary studies demonstrate pretreatment group equivalence via matching, statistical controls, or evidence of equivalence on key risk variables and participant characteristics as outlined in Section 3.3.4.

#### 3.3.2 Selection of studies

Under the supervision of review authors, two review team assistants will first independently screen titles and abstracts to exclude studies that are clearly irrelevant. Studies considered eligible by at least one assistant or studies were there is insufficient information in the title and abstract to judge eligibility, will be retrieved in full text. The full texts will then be screened independently by two review team assistants under the supervision of the review authors. Any disagreement of eligibility will be resolved by the review authors. Exclusion reasons for studies that otherwise might be expected to be eligible will be documented and presented in an appendix.

The study inclusion criteria will be piloted by the review authors and team assistants (see Supporting Information: Appendix 2). The overall search and screening process will be illustrated in a flow diagram. None of the review team members will be blind to the authors, institutions, or the journals responsible for the publication of the articles.

#### 3.3.3 Data extraction and management

Two review authors will independently code and extract data from included studies. A coding sheet will be piloted on several studies and revised as necessary (see Supporting Information: Appendix 3). Disagreements will be resolved by consulting a third review author with extensive content and methods expertise. Disagreements resolved by a third reviewer will be reported. Data and information will be extracted on: available characteristics of participants, intervention characteristics and control conditions, research design, sample size, risk of bias and potential confounding factors, outcomes, and results. Extracted data will be stored electronically. Analysis will be conducted using RevMan5 and Stata software.

#### 3.3.4 Assessment of risk of bias in included studies

We will assess the risk of bias in randomised studies using Cochrane's revised risk of bias tool, ROB 2 (Higgins et al., 2019).

The tool is structured into five domains, each with a set of signalling questions to be answered for a specific outcome. The five domains cover all types of bias that can affect results of randomised trials.

The five domains for individually randomised trials are:

(1) bias arising from the randomisation process;
(2) bias due to deviations from intended interventions (separate signalling questions for effect of assignment and adhering to intervention);
(3) bias due to missing outcome data;
(4) bias in measurement of the outcome;
(5) bias in selection of the reported result.

For cluster‐randomised trials, an additional domain is included ((1b) Bias arising from identification or recruitment of individual participants within clusters). We will use the latest template for completion (currently it is the version of 15 March 2019 for individually randomised parallel‐group trials and 20 October 2016 for cluster randomised parallel‐group trials). In the cluster randomised template however, only the risk of bias due to deviation from the intended intervention (effect of assignment to intervention; intention to treat ITT) is present and the signalling question concerning the appropriateness of the analysis used to estimate the effect is missing. Therefore, for cluster randomised trials we will only use the signalling questions concerning the bias arising from identification or recruitment of individual participants within clusters from the template for cluster randomised parallel‐group trials; otherwise we will use the template and signalling questions for individually randomised parallel‐group trials.

We will assess the risk of bias in non‐randomised studies, using the model ROBINS –I, developed by members of the Cochrane Bias Methods Group and the Cochrane Non‐Randomised Studies Methods Group (Sterne, Hernán, et al., 2016). We will use the latest template for completion (currently it is the version of 19 September 2016).

The ROBINS‐I tool is based on the Cochrane RoB tool for randomised trials, which was launched in 2008 and modified in 2011 (Higgins et al., 2011).

The ROBINS‐I tool covers seven domains (each with a set of signalling questions to be answered for a specific outcome) through which bias might be introduced into non‐randomised studies:

(1) bias due to confounding
(2) bias in selection of participants
(3) bias in classification of interventions
(4) bias due to deviations from intended interventions;
(5) bias due to missing outcome data;
(6) bias in measurement of the outcome;
(7) bias in selection of the reported result.

The first two domains address issues before the start of the interventions and the third domain addresses classification of the interventions themselves. The last four domains address issues after the start of interventions and there is substantial overlap for these four domains between bias in randomised studies and bias in non‐randomised studies trials (although signalling questions are somewhat different in several places, see Sterne, Higgins, et al., 2016 and Higgins et al., 2019).

Randomised study outcomes are rated on a ‘Low/Some concerns/High’ scale on each domain; whereas non‐randomised study outcomes are rated on a ‘Low/Moderate/Serious/Critical/No Information’ scale on each domain. The level ‘Critical’ means: the study (outcome) is too problematic in this domain to provide any useful evidence on the effects of intervention, and it is excluded from the data synthesis. The same critical level of risk of bias (excluding the result from the data synthesis) is not directly present in the RoB 2 tool, according to the guidance to the tool (Higgins et al., 2019).

In the case of a RCT, where there is evidence that the randomisation has gone wrong or is no longer valid, we will assess the risk of bias of the outcome measures using ROBINS‐I instead of ROB 2. Examples of reasons for assessing RCTs using the ROBINS‐I tool may include studies showing large and systematic differences between treatment conditions while not explaining the randomisation procedure adequately suggesting that there was a problem with the randomisation process; studies with large‐scale differential attrition between conditions in the sample used to estimate the effects; or studies selectively reporting results for some part of the sample or for only some of the measured outcomes. In such cases, differences between the treatment and control conditions are likely systematically related to other factors than the intervention and the random assignment is, on its own, unlikely to produce unbiased estimates of the intervention effects. Therefore, as ROBINS‐I allow for an assessment of for example confounding, we believe it is more appropriate to assess effect sizes from studies with a compromised randomisation using ROBINS‐I than ROB 2. If so, we will report this decision as part of the risk of bias assessment of the outcome measure in question. As other effect sizes assessed with ROBINS‐I, these effect sizes may receive a ‘Critical’ rating and thus be excluded from the data synthesis.

We will stop the assessment of a non‐randomised study outcome as soon as one domain in the ROBINS‐I is judged as ‘Critical’.

‘Serious’ risk of bias in multiple domains in the ROBINS‐I assessment tool may lead to a decision of an overall judgement of ‘Critical’ risk of bias for that outcome, and it will be excluded from the data synthesis.

##### Confounding

An important part of the risk of bias assessment of non‐randomised studies is consideration of how the studies deal with confounding factors. Systematic baseline differences between groups can compromise comparability between groups. Baseline differences can be observable (e.g., age and gender) and unobservable (to the researcher; e.g., motivation and ‘ability’). There is no single non‐randomised study design that always solves the selection problem. Different designs represent different approaches to dealing with selection problems under different assumptions, and consequently require different types of data. There can be particularly great variations in how different designs deal with selection on unobservables. The ‘adequate’ method depends on the model generating participation, that is, assumptions about the nature of the process by which participants are selected into a programme.

A major difficulty in estimating causal effects of sport participation is the potential endogeneity of the young individual's life circumstance and developmental state that leads to the decision of participating in organised sport and if not accounted for it will yield biased estimates.

As there is no universal correct way to construct counterfactuals for non‐randomised designs, we will look for evidence that identification is achieved, and that the authors of the primary studies justify their choice of method in a convincing manner by discussing the assumption(s) leading to identification (the assumption(s) that make it possible to identify the counterfactual). Preferably the authors should make an effort to justify their choice of method and convince the reader that the only difference between a treated individual and a non‐treated individual is the treatment. The judgement is reflected in the assessment of the confounder unobservables in the list of confounders considered important at the outset (see Supporting Information: Appendix 4).

In addition to unobservables, we have identified the following observable confounding factors to be most relevant: age, gender and risk indicators as described in Section 3.1.2. In each study, we will assess whether these indicators have been considered, and in addition we will assess other factors likely to be a source of confounding within the individual included studies.

##### Importance of pre‐specified confounding factors

The motivation for focusing on age, gender and risk indicators is given below.

The prevalence of different types of behavioural and psychological problems, coping skills, cognitive and emotional ability vary throughout a child's development through puberty and into adulthood (Cole et al., 2005), and therefore we consider age to be a potential confounding factor. Furthermore, there are substantial gender differences in behaviour problems, coping and risk of different types of adverse outcomes which is why we also include gender as a potential confounding factor (Card et al., 2008; Hampel & Petermann, 2005; Hart et al., 2007).

Pre‐treatment group equivalence of risk indicators is indisputable an important confounder as young people in stable life circumstances, typically are not at risk of developing the range of problems we will consider in this review. Therefore, the accuracy of the estimated effects of sport programmes will depend crucially on how well the risk indicators are controlled for.

##### Effect of primary interest and important co‐interventions

We are mainly interested in the effect of starting and adhering to the intended intervention, that is, the treatment on the treated (TOT) effect. The risk of bias assessments will therefore be in relation to this specific effect.

The risk of bias assessments of both randomised trials and non‐randomised studies will consider adherence and differences in additional interventions (‘co‐interventions’) between intervention groups. Relevant co‐interventions are those that individuals might receive with or after starting the intervention of interest and that are both related to the intervention received and prognostic for the outcome of interest. Important co‐interventions we will consider are interventions delivered as part of sport‐based youth development programmes. These programmes may be explicitly teaching personal and social responsibility or other life skills. Although these types of programmes are not eligible (see Section 3.1.3) we will carefully consider if there are any co‐interventions teaching other than the sport discipline in question.

##### Assessment

At least two review authors will independently assess the risk of bias for each relevant outcome from the included studies. Any disagreements will be resolved by a third reviewer with content and statistical expertise and will be reported. We will report the risk of bias assessment in risk of bias tables for each included study outcome in the completed review.

#### 3.3.5 Measures of treatment effect

##### Continuous outcomes

For continuous outcomes, effects sizes with 95% confidence intervals will be calculated, where means and standard deviations are available. If means and standard deviations are not available, we will calculate standardised mean differences (SMDs) from *F*‐ratios, *t*‐values, *χ* ^2^ values and correlation coefficients, where available, using the methods suggested by Lipsey and Wilson (2001). If not enough information is yielded, the review authors will request this information from the principal investigators. Hedges’ *g* will be used for estimating SMD. Any measures of drug and alcohol use or social and emotional outcomes, are examples of relevant continuous outcomes in this review.

##### Dichotomous outcomes

For dichotomous outcomes, we will calculate odds ratios with 95% confidence intervals. Delinquency and school failure, are examples of relevant dichotomous outcomes in this review.

There are statistical approaches available to re‐express dichotomous and continuous data to be pooled together (Sánchez‐Meca et al., 2003). To calculate common metric odds ratios will be converted to SMD effect sizes using the Cox transformation. We will only transform dichotomous effect sizes to SMD if appropriate, for example, as may be the case with for example the outcomes drug and alcohol use, that can be measured with binary and continuous data.

When effect sizes cannot be pooled, study‐level effects will be reported in as much detail as possible. Software for storing data and statistical analyses will be RevMan 5.0, Excel, R and Stata 10.0.

#### 3.3.6 Unit of analysis issues

Errors in statistical analysis can occur when the unit of allocation differs from the unit of analysis. In cluster randomised trials, participants are randomised to treatment and control groups in clusters, either when data from multiple participants in a setting are included (creating a cluster within the community setting), or when participants are randomised by treatment locality. Non‐randomised studies may also include clustered assignment of treatment. Effect sizes and standard errors from such studies may be biassed if the unit‐of‐analysis is the individual and an appropriate cluster adjustment is not used (Higgins & Green, 2011).

A study design where participants are individually allocated to treatment, but the treatment is delivered in a group setting, are known as *individually randomised group treatment* (IRGT) trials (Pals et al., 2008). The analysis in such a study design must also correct for the fact that dependencies may arise between individuals that happen to receive the intervention in the same group.

If possible, we will adjust effect sizes individually using the methods suggested by Hedges (2007b) and information about the intra‐cluster correlation coefficient (ICC), realised cluster sizes, and/or estimates of the within and between variances of clusters. If it is not possible to obtain this information, we will adjust effect sizes using estimates from the literature (we will search for estimates of relevant ICCs), and assume equal cluster sizes. To calculate an average cluster size, we will divide the total sample size in a study by the number of clusters.

#### 3.3.7 Criteria for determination of independent findings

To determine the independence of results in included studies, we will consider whether individuals may have undergone multiple interventions, whether there were multiple treatment groups, whether several studies are based on the same data source and whether studies report multiple conceptually similar outcomes.

##### Multiple interventions groups and multiple interventions per individuals

Studies with multiple intervention groups with different individuals will be included in this review, although only intervention and control groups that meet the eligibility criteria will be used in the data synthesis. To avoid problems with dependence between effect sizes we will apply robust standard errors (Hedges et al., 2010) and use the small sample adjustment to the estimator itself (Tipton, 2015). We will use the results in Tanner‐Smith and Tipton (2014); and Tipton (2015) to evaluate if there are enough studies for this method to consistently estimate the standard errors. See Section 3.3.11 for more details about the data synthesis.

If there are not enough studies, we will use a synthetic effect size (the average) to avoid dependence between effect sizes. This method provides an unbiased estimate of the mean effect size parameter but overestimates the standard error. Random effects models applied when synthetic effect sizes are involved actually perform better in terms of standard errors than do fixed effects models (Hedges, 2007a). However, tests of heterogeneity when synthetic effect sizes are included are rejected less often than nominal.

If pooling is not appropriate (e.g., the multiple interventions and/or control groups include the same individuals), only one intervention group will be coded and compared to the control group to avoid overlapping samples. The choice of which estimate to include will be based on our risk of bias assessment. We will choose the estimate that we judge to have the least risk of bias (primarily, Confounding bias and in case of equal scoring the Missing outcome data domain will be used).

##### Multiple studies using the same sample of data

In some cases, several studies may have used the same sample of data or some studies may have used only a subset of a sample used in another study. We will review all such studies, but in the meta‐analysis we will only include one estimate of the effect from each sample of data. This will be done to avoid dependencies between the ‘observations’ (i.e., the estimates of the effect) in the meta‐analysis. The choice of which estimate to include will be based on our risk of bias assessment of the studies. We will choose the estimate from the study that we judge to have the least risk of bias (primarily, Confounding bias). If two (or more) studies are judges to have the same risk of bias and one of the studies (or more) uses a subset of a sample used in another study (or studies) we will include the study using the full set of participants.

##### Multiple time points

When the results are measured at multiple time points, each outcome at each time point will be analysed in a separate meta‐analysis with other comparable studies taking measurements at a similar time point. As a general guideline, these will be grouped together as follows: (1) post‐intervention, (2) less than a year follow up, (3) 1–2 year follow up and (4) More than 2 year follow up. However, should the studies provide viable reasons for an adjusted choice of relevant and meaningful duration intervals for the analysis of outcomes, we will adjust the grouping.

##### Multiple conceptually similar outcomes

Meta‐analysis of outcomes will be conducted on each metric (as outlined in Section 3.1.4) separately. If there are multiple estimates of effects regarding the same/similar outcome (e.g., externalising behaviour measured with the Child Behavior Checklist subscale or the Strengths and Difficulties Questionnaire subscale), we will extract (and report) all outcomes, but in the meta‐analysis we will include the measure which most closely match the outcomes used in the other studies included in that particular meta‐analysis.

#### 3.3.8 Dealing with missing data

Missing data and attrition rates will be assessed in the included studies; see Section 3.3.4. Where studies have missing summary data, such as missing standard deviations, the review authors will request this information from the principal investigators. If no information is yielded, we will derive these where possible from *F*‐ratios, *t*‐values, *χ* ^2^ values and correlation coefficients using the methods suggested by Lipsey and Wilson (2001). If missing summary data cannot be derived, the study results will be reported in as much detail as possible.

#### 3.3.9 Assessment of heterogeneity

Heterogeneity amongst primary outcome studies will be assessed with *χ* ^2^ (*Q*) test, and the *I* ^2^, and *τ* ^2^ statistics (Higgins et al., 2003). Any interpretation of the *χ* ^2^ will be made cautiously on account of its low statistical power.

#### 3.3.10 Assessment of reporting biases

Reporting bias refers to both publication bias and selective reporting of outcome data and results. Here, we state how we will assess publication bias.

We will use funnel plots for information about possible publication bias if we find sufficient studies (Higgins & Green, 2011). However, asymmetric funnel plots are not necessarily caused by publication bias (and publication bias does not necessarily cause asymmetry in a funnel plot). In general, asymmetry is a sign of small‐study effects, of which there can be many causes beside publication bias (Sterne et al., 2005).

Instead of trying to interpret the funnel plots as direct evidence of publication bias, or the lack thereof, we will perform sensitivity analyses for publication bias in meta‐analyses as suggested by Mathur & VanderWeele, 2020. This method gives a value of how large ratios of publication probabilities (i.e., the likelihood of affirmative results to be published relative to non‐affirmative results) would have to be to alter the results and therefore indicate how robust the meta‐analysis is to publication bias.

#### 3.3.11 Data synthesis

The proposed project will follow standard procedures for conducting systematic reviews using meta‐analysis techniques.

All follow‐up durations reported in the primary studies will be recorded, and we will do separate analyses for short‐term and long‐term outcomes.

The overall data synthesis will be conducted where effect sizes are available or can be calculated, and where studies are similar in terms of the outcome measured. Meta‐analysis of outcomes will be conducted on each metric (as outlined in Section 3.1.4) separately.

Analysis of absolute effects (comparing sport participation to no treatment) and relative effects (comparing sport participation to an alternative treatment) will be conducted separately.

As different computational methods may produce effect sizes that are not comparable, we will be transparent about all methods used in the primary studies (research design and statistical analysis strategies) and use caution when synthesising effect sizes. Special caution will be taken concerning studies using regression discontinuity designs (RDD) to estimate the treatment effect. In sharp RDDs, a threshold of a (non‐manipulable) forcing/running variable determines which students receive a treatment and which do not, that is, the design is similar to a RCT in the sense that the random sequence determining treatment assignment can be seen as a running variable (Lee & Lemieux, 2010). In contrast, in ‘fuzzy’ RDDs, being on one side of a threshold is a special type of IV only makes it more likely that a student end up in the treatment or control group, and the threshold is used as an instrument to estimate local average treatment effects (LATE) (Angrist & Pischke, 2009; Imbens & Lemieux, 2008). That is, fuzzy RDD is a form of IV analysis, which we will exclude due to the comparability issues mentioned earlier. Sharp RDDs will be included, but, as the effects may be estimated on a very ‘local’ sample close to a threshold, may be subject to a separate analysis depending on the comparability to samples from other studies. We will in any case check the sensitivity of our results to the inclusion of RDD studies. In addition, we will discuss the limitation in generalisation of results obtained from these types of studies.

When the effect sizes used in the data synthesis are odds ratios, they will be log transformed before being analysed. The reason is that ratio summary statistics all have the common feature that the lowest value that they can take is 0, that the value 1 corresponds with no intervention effect, and the highest value that an odds ratio can ever take is infinity. This number scale is not symmetric. The log transformation makes the scale symmetric: the log of 0 is minus infinity, the log of 1 is zero, and the log of infinity is infinity.

Studies that have been coded with a Critical risk of bias will not be included in the data synthesis.

As the intervention deal with diverse populations of participants (from different countries, facing different life circumstances, etc.), and we therefore expect heterogeneity amongst primary study outcomes, all analyses of the overall effect will be inverse variance weighted using random effects statistical models that incorporate both the sampling variance and between study variance components into the study level weights. Random effects weighted mean effect sizes will be calculated using 95% confidence intervals, and we will provide a graphical display (forest plot) of effect sizes. Graphical displays for meta‐analysis performed on ratio scales sometimes use a log scale, as the confidence intervals then appear symmetric. This is however not the case for the software Revman 5 which we plan to use in this review (If we apply robust variance estimation, the analysis will be conducted in Stata or R as robust variance estimation is not implemented in Revman 5). The graphical displays using odds ratios and the mean effect size will be reported as an odds ratio. Heterogeneity amongst primary outcome studies will be assessed with *χ* ^2^ (*Q*) test, and the *I* ^2^, and *τ* ^2^ statistics (Higgins et al., 2003). Any interpretation of the *χ* ^2^ test will be made cautiously on account of its low statistical power.

95% prediction intervals will be reported.

For subsequent analyses of moderator variables that may contribute to systematic variations, we will use the mixed‐effects regression model if there are a sufficient number of studies. This model is appropriate if a predictor explaining some between‐studies variation is available, but there is a need to account for the remaining uncertainty (Hedges & Pigott, 2004; Konstantopoulos, 2006).

Several studies may have used the same sample of data. We will review all such studies, but in the meta‐analysis we will only include one estimate of the effect from each sample of data. This will be done to avoid dependencies between the ‘observations’ (i.e. the estimates of the effect) in the meta‐analysis. The choice of which estimate to include will be based on our quality assessment of the studies. We will choose the estimate from the study that we judge to have the least risk of bias, with particular attention paid to Confounding bias.

Studies may only provide results separated by for example age and/or gender and not for the overall sample. We will include results for all age and gender groups. To take into account the dependence between such multiple effect sizes from the same study, we will apply robust variance estimation (RVE) approach (Hedges et al., 2010). An important feature of this analysis is that the results are valid regardless of the weights used. For efficiency purposes, we will calculate the weights using a method proposed by Hedges et al. (2010). This method assumes a simple random‐effects model in which study average effect sizes vary across studies (*τ* ^2^) and the effect sizes within each study are equi correlated (*ρ*). The method is approximately efficient, since it uses approximate inverse‐variance weights: they are approximate given that *ρ* is, in fact, unknown and the correlation structure may be more complex. We will calculate weights using estimates of *τ* ^2^, setting *ρ* = 0.80 and conduct sensitivity tests using a variety of *ρ* values; to assess if the general results and estimates of the heterogeneity is robust to the choice of *ρ*. We will use the small sample adjustment to the residuals used in RVE as proposed by Bell and McCaffrey (2002) and extended by McCaffrey et al. (2001) and by Tipton (2015). We will use the Satterthwaite degrees of freedom (Satterthwaite, 1946) for tests as proposed by Bell and McCaffrey (2002) and extended by Tipton (2015). We will use the guidelines provided in Tanner‐Smith and Tipton (2014) to evaluate if there are enough studies for this method to consistently estimate the standard errors.

If there is not a sufficient number of studies to use RVE we will conduct a data synthesis where we use a synthetic effect size (the average) to avoid dependence between effect sizes.

#### 3.3.12 Subgroup analysis and investigation of heterogeneity

We will investigate the following factors with the aim of explaining potential observed heterogeneity: study‐level summaries of participant characteristics (e.g., studies considering a specific gender or age group or studies where separate effects for girls/boys or age groups (e.g., 6–12 year old/13–18 year old) are available) and risk indicator (e.g., studies considering a specific risk indicator or studies where separate effects for low socioeconomic status or delinquent youth are available). In addition, we will investigate programme characteristics such as team/individual, contact/no contact, intensity and duration.

If the number of included studies is sufficient and given there is variation in the covariates (age, gender, risk indicator and programme characteristics), we will perform moderator analyses (multiple meta‐regression using the mixed model) to explore how observed variables are related to heterogeneity.

If there are a sufficient number of studies, we will apply the RVE approach and use approximately inverse variance weights calculated using a method proposed by Hedges et al. (2010). This technique calculates standard errors using an empirical estimate of the variance: it does not require any assumptions regarding the distribution of the effect size estimates. The assumptions that are required to meet the regularity conditions are minimal and generally met in practice. This more robust technique is beneficial because it takes into account the possible correlation between effect sizes separated by the covariates within the same study (e.g., age or gender separated effects) and allows all the effect size estimates to be included in meta‐regression. We will calculate weights using estimates of *τ* ^2^, setting *ρ* = 0.80 and conduct sensitivity tests using a variety of *ρ* values; to assess if the general results are robust to the choice of *ρ*. We will use the small sample adjustment to the residuals used in RVE and the Satterthwaite degrees of freedom (Satterthwaite, 1946) for tests (Tipton, 2015). The results in Tipton (2015) suggests that the degrees of freedom depend on not only the number of studies but also on the type of covariates included in the meta‐regression. The degrees of freedom can be small, even when the number of studies is large if a covariate is highly unbalanced or a covariate with very high leverage is included, The degrees of freedom will vary from coefficient to coefficient. The corrections to the degrees of freedom enable us to assess when the RVE method performs well. As suggested by Tanner‐Smith and Tipton (2014) and Tipton (2015) if the degrees of freedom are smaller than four, the RVE results should not be trusted.

We will report 95% confidence intervals for regression parameters. We will estimate the correlations between the covariates and consider the possibility of confounding. Conclusions from meta‐regression analysis will be cautiously drawn and will not solely be based on significance tests. The magnitude of the coefficients and width of the confidence intervals will be taken into account as well. Otherwise, single factor subgroup analysis will be performed. The assessment of any difference between subgroups will be based on 95% confidence intervals. Interpretation of relationships will be cautious, as they are based on subdivision of studies and indirect comparisons.

In general, the strength of inference regarding differences in treatment effects amongst subgroups is controversial. However, making inferences about different effect sizes amongst subgroups on the basis of between‐study differences entails a higher risk compared to inferences made on the basis of within study differences; see Schandelmaier et al. (2020). We will therefore use within study differences where possible.

We will also consider the degree of consistence of differences, as making inferences about different effect sizes amongst subgroups entails a higher risk when the difference is not consistent within the studies; see Schandelmaier et al. (2020).

#### 3.3.13 Sensitivity analysis

Sensitivity analysis will be carried out by restricting the meta‐analysis to a subset of all studies included in the original meta‐analysis and will be used to evaluate whether the pooled effect sizes are robust across components of risk of bias. We will consider sensitivity analysis for each domain of the risk of bias checklists and restrict the analysis to studies with a low risk of bias.

Sensitivity analyses with regard to research design and statistical analysis strategies in the primary studies will be an important element of the analysis to ensure that different methods produce consistent results.

#### 3.3.14 Treatment of qualitative research

We do not plan to include qualitative research.

#### 3.3.15 Summary of findings and assessment of the certainty of the evidence

We do not plan to include Summary of findings and assessment of the certainty of the evidence.

## PRELIMINARY TIMEFRAME

Approximate date for submission of the systematic review will be no longer than 2 years after protocol approval.

## PLANS FOR UPDATING THIS REVIEW

Trine Filges will be responsible for updating the review and updates can be expected each second year.

## CONTRIBUTIONS OF AUTHORS

- Content: Else Ladekjær, Trine Filges
- Systematic review methods: Trine Filges
- Statistical analysis: Trine Filges, Mette Verner
- Information retrieval: Elizabeth Bengtsen

## DECLARATIONS OF INTEREST

There are no potential conflicts of interest.

## SOURCES OF SUPPORT

**Internal sources**

- No sources of support provided

**External sources**

- No sources of support provided

## Supporting information

Supporting information.
